# Improving Plastic Dressing Clinic Burden by Reducing Punch Biopsy Referrals Using a Patient Information Leaflet: A Quality Improvement Project

**DOI:** 10.7759/cureus.30999

**Published:** 2022-11-02

**Authors:** Sipan Shahnazari

**Affiliations:** 1 Surgery, Imperial College London, London, GBR

**Keywords:** skin cancer, patient information, punch biopsy, quality improvement, leaflet

## Abstract

Background

Although being a safe intervention with low complication rates, a significant proportion of patients undergoing punch biopsies were referred to the local plastic dressing clinic (PDC) despite providing minimal utility and adding to an already heavily burdened clinic. The aim of this quality improvement (QI) project was to reduce the number of patients who had undergone a punch biopsy who were referred to PDC. The steering group included a plastic surgeon consultant, a senior outpatient department (OPD) and a PDC nurse, and a junior trainee. Process mapping and driver diagrams were used to identify a patient information leaflet as the intervention.

Methods

A patient information leaflet was created by the clinical lead, which included advice for postoperative wound care and when to seek medical attention. This leaflet was provided to every patient who underwent a minor operation for a skin lesion from September 2021. Operative notes of patients who underwent a punch biopsy were reviewed from October to November 2021 and from August to September 2022 to identify the number of patients who were referred to PDC. Concurrently informal feedback from patients and the process manager guided any necessary changes.

Results

There was a small improvement in the number of PDC referrals during October-November 2021 (46%) compared to the baseline measurements (54%). This corresponded with an absolute risk reduction (ARR) of 7%, risk ratio (RR) of 0.86, and relative risk reduction (RRR) of 14%. No changes were made at this stage. There was a much greater improvement in August-September 2022 (20%) compared to the baseline measurements with 34% ARR, 0.37 RR, and 63% RRR. Informal feedback from the process manager confirmed that every patient received a leaflet and that the patients found the leaflet very helpful.

Conclusions

The implementation of the patient information leaflet has led to a reduction in referrals of patients with punch biopsies to PDC. While this is a small and narrow project, it demonstrates the values of QI methodology to select a successful intervention. Furthermore, it corroborates with other studies that have shown that patient information leaflets empower patients and can help reduce the National Health Service (NHS) outpatient burden. Considering its simplicity, trialing a similar approach in other specialties is strongly encouraged.

## Introduction

A punch biopsy is a frequently used intervention for skin lesion diagnostics. It is a low-risk modality with less than 1% causing complications such as postoperative bleeding, wound infection, and surrounding skin damage [1]. Because of this, many dermatology units across the United Kingdom (UK) do not review the punch biopsy wound postoperatively but instead provide information leaflets and advise primary care review or contacting the secondary care nursing team if there are any concerns [2,3].

The plastic and reconstructive surgery department at Charing Cross Hospital, Imperial College Healthcare Trust (ICHT), is one of the busiest complex reconstructive services in the country performing over 250 reconstructions for breast, head, neck, and skin cancers annually [4]. Following discharge, these patients’ wounds are reviewed by a nurse in the outpatient plastic dressing clinic (PDC). This service is provided every weekday by a team of three clinical nurse specialists (CNS). Beyond these duties, the CNS team also reviews patients who have undergone a minor procedure for skin lesions.

A survey of the PDC team in August 2021 revealed that PDC overran on most days, with the nurse feeling rushed when seeing patients, and that this could be reduced by minimizing the number of reviews for minor operations including punch biopsies and direct closure. Indeed, a retrospective review of the operation notes of patients from October to November 2020 demonstrated that of the 28 patients who underwent a punch biopsy only, 15 were referred to PDC for postoperative review (54%). None had any complications, and all were discharged from PDC.

Rationale

To identify an optimal intervention to resolve this issue, a process map (Appendix 1) was created to understand the patient journey from intervention to PDC. It was found that all patients who had undergone a complex reconstruction had a PDC review within seven days. For this cohort, the PDC is of high value for clinicians and patients and therefore cannot be altered.

However, whether a patient who had undergone a minor operation for a skin lesion was recalled to PDC was entirely at the surgeons’ discretion with no uniform approach. Furthermore, all of these patients received a telephone follow-up from the surgical team to discuss histopathology results and review their wound healing. Thus, there was duplication of work because all patients underwent a telephone review of their wounds whether or not they had been reviewed by PDC. Therefore, it was felt that the Lean methodology could be used to simplify this part of the patient journey [5].

A driver diagram (Appendix 2) identified change ideas most likely to achieve the aim of reducing punch biopsy wound review in PDC [6]. It was quickly identified that a number of change ideas (e.g., PDC contact details and patient and surgeon education) could all be integrated into one intervention as a patient information leaflet. Using a Kaizen approach, we believed that this small change could lead to an incremental improvement [7].

Finally, a stakeholder analysis (Appendix 3) was undertaken to identify the key individuals for decision-making and implementation. The resulting priority of engagement identified plastic surgeon consultants, PDC CNS, and outpatient department (OPD) CNS as required cocreators. Therefore, a consultant plastic became the sponsor and clinical expert, the senior CNS of OPD became the process owner, and a junior trainee became the project leader. Based on the above, the group decided that a patient information leaflet had the strongest opportunity for success.

Specific aims

The aim of this quality improvement (QI) project is to reduce the number of punch biopsy wounds reviewed at the plastic dressing clinic (PDC) through the use of a patient information leaflet within one year. Both outcomes and processes will be assessed in order to demonstrate the adoption of the desired practice.

## Materials and methods

Context

ICHT has a well-established QI department (“Helping Our Teams Transform” (HOTT)), which encourages such projects. There was strong buy-in from the sponsor, clinical expert, process owner, and project leader who had training in QI methodology. The Model of Understanding Success in Quality (MUSIQ) score was calculated to be 134, revealing that the project had a reasonable chance of success [8,9]. Key weaknesses were the lack of external incentives and motivators to undertake this QI project.

Intervention

A patient information leaflet was created by the sponsor and clinical expert. The information included whether the sutures were absorbable, when a wound review was needed by their general practitioner or PDC, common postoperative complaints (swelling and bruising), how to manage these, advice regarding soaking and cleaning, and the risks of exercise, wound infection, and smoking. Furthermore, contact details were provided for the PDC nurse. It, therefore, addressed the common concerns of the patients.

The leaflet was provided to every patient undergoing a minor operation for a skin lesion. In order to keep the change sustainable, the leaflet became part of the print-off package produced by the nursing staff prior to a patient undergoing a skin excision. Thereby, the process system was changed directly [10].

It was believed that the patient information leaflet would work because it simultaneously empowered the patients and assured the surgeon that they had the knowledge they required to appropriately review their wounds safely. We hypothesized that this would lead to a reduction in the number of patients referred to PDC following a punch biopsy (Table 1) [11].

**Table 1:** Patient information leaflet described as per the Template for Intervention Description and Replication (TIDIeR) checklist PDC: plastic dressing clinic, WHO: World Health Organization, OPD: outpatient department, CNS: clinical nurse specialist, ICHT: Imperial College Healthcare Trust

Brief name	Postoperative information for patient leaflet
Why	Small intervention covering key change ideas to reduce PDC reviews
What	Materials: double-sided A4 physical printout; process: leaflet is printed as part of patient preoperative printout package, which includes the WHO checklist and OPD flow sheet
Who	The patient leaflet was written by a plastic surgeon consultant, and implementation was achieved by a CNS in charge of the OPD
How	The patient is given a leaflet prior to leaving the minor operation room after their procedure
Where	Printing took place in the nursing station preoperatively as part of the patient printout package; the patient brings all printouts to the minor operations room
When and how much	The patient leaflet was implemented in September 2021 for all patients undergoing a minor operation
Tailoring	Reviewed by the ICHT communications team; no adjustments needed
Modifications	None

Study of intervention

A quasi-experimental evaluation study design was used to determine whether the patient information leaflet reduced the number of patients with punch biopsies who were reviewed in PDC. Data extraction occurred once before the implementation of the information leaflet and twice after.

Measures

The measure chosen for studying the outcomes of the intervention was the proportion of patients who had undergone a punch biopsy whose postoperative plan included PDC review as per the operative note. Patients who had also had another procedure during the same operation were excluded. The number of punch biopsies per patient was not collected, only whether they were referred to PDC. The operative note was used because it most accurately depicts the instructions given by the operator to the patient. This indicates the extent to which the operators believe a PDC review is indicated. There was a preliminary phase for baseline measurement and then two subsequent data collections.

Analysis

Absolute risk (AR), absolute risk reduction (ARR), relative risk (RR), and relative risk reduction (RRR) were used to demonstrate a reduction in patients being referred to PDC and the size of this effect. A run chart was compiled in order to look for shifts, trends, anomalies, and runs between the percentage of referrals to PDC per week. A control chart could not be made as there was too much variation in the data, causing the standard deviation to be too high.

Ethical considerations

Approval for this study was provided by the Imperial College Audit team. This study does not meet the threshold of research requiring ethical approval as per the National Health Service (NHS) Health Research Authority Research Toolkit [12].

## Results

The first phase of data collection was to review whether our leaflet had made a difference. This took place during October and November 2021. There was a small reduction in the proportion of patients who were being referred to PDC (46% AR, 7% ARR, 0.86 RR, and 14% RRR). There were no shifts or trends on the run chart. The percentage of patients with punch biopsies who were referred to PDC was anomalous in week 6. Discussions with the process owner revealed that the process was fully functional with every patient undergoing a minor procedure receiving the patient leaflet as they left. There was good feedback from the theater nurses who felt empowered by the clarity the patient leaflet provided the patients. It was decided that no further changes were to be made to the intervention or the process.

**Figure 1:**
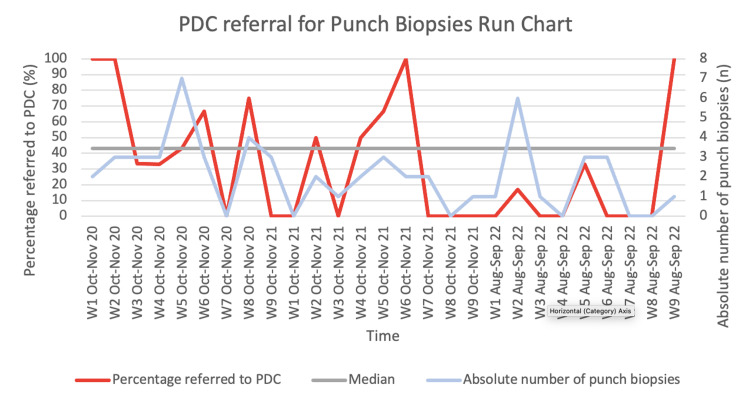
Run chart of PDC referrals for punch biopsies PDC: plastic dressing clinic

The second phase took place between August and September 2022. Compared to the baseline measurements, there was a substantially greater reduction in patients referred to PDC (20% AR, 34% ARR, 0.37 RR, and 63% RRR). This correlated to the run chart (Figure 1), which demonstrated a downward shift from the first week of August. There was an anomalous final result of 100% referral to PDC in the last week. Across the course of the entire run chart, the number of runs was within expectation. The process owner reconfirmed that there were no issues with the process of providing the leaflet.

Informal verbal feedback from the patients found that the leaflet relieved anxiety about postoperative care, was informative regarding what to avoid and what to look out for, and was a helpful reference for whom to contact.

There was no missing data during the course of this study.

## Discussion

Summary

In this study, we were able to demonstrate a reduction in patients who had undergone a punch biopsy and were referred to the PDC from 54% to 20%. We were able to achieve this by undertaking a deep dive into the process we had in place through process mapping, identifying key stakeholders and actors who could drive the project, and identifying a simple intervention that could address most of the change ideas sustainably.

We believe that the intervention worked because there were no other changes regarding education provided to the surgeons or any other change in the process. Indeed, it is likely that we have introduced a cultural shift in the minor operations department as referrals to PDC in the second data collection were consistently low. This is similarly supported by the finding that there was minimal improvement soon after the intervention was released. Therefore, it is not solely the information leaflet that brings about the change but the confidence of the surgeon in the patients’ ability to care for their wounds. Paramount to achieving this aim was ensuring that the process was kept consistent since implementation such that all patients received the leaflet in a standardized manner.

Our study correlates with others in that patients approve of the use of patient information leaflets. There are other benefits received by patients with information leaflets. A similar patient information leaflet at the dermatology department in Swindon demonstrated that patients find the information leaflets very helpful [13]. Furthermore, it has been shown that the implementation of a patient information leaflet can reduce the burden on NHS services. Using a patient information leaflet, Bonfield was able to demonstrate a reduction in readmission rates for patients who had had an acute kidney injury (AKI) during their prior admission [14]. Therefore, the power of patient information leaflets should not be understated in their ability to reduce the NHS burden by informing the patient population and should be used widely.

Although there are financial and environmental costs to such interventions, it is likely that these are greatly offset by their benefits for the NHS and patients. It serves to reduce unnecessary outpatient department visits and thus contributes to the NHS Long Term Plan to reduce outpatient visits by 30 million a year and save £1 billion annually [15]. Similarly, it reduces hidden harm to patients of unnecessary hospital appointments, including the stress of traveling, time wasted, and contribution to deaths from traveling (753 deaths annually due to contribution to air pollution and 85 deaths annually due to accidents) [16]. Patient leaflets, therefore, provide means of reducing the NHS outpatient burden.

Limitations

There are a number of limitations to the project. The first is the lack of involvement of the patient population from the inception of the project. Through the use of semi-structured interviews and focus groups, it would have been possible to be certain that the information leaflet addressed their issues. Similarly, assessing patient-related outcome measures (PROMs) following the start of the intervention was not done formally. Opportunities were, therefore, likely missed regarding the improvement of the leaflet and identifying whether the leaflet was the cause of the improved rate of PDC referral. This is something we will take forward into future projects. However, by receiving feedback informally, we were confident that the intervention was working well.

Similarly, we did not collect quantitative data surrounding our process measure, i.e., collecting data regarding the number of patients who received the patient leaflet. This could have been done by having a separate assessor ask patients if they had received a leaflet at random time intervals. However, informal reporting from the multidisciplinary team demonstrated that almost all patients were receiving the leaflet.

Another issue is the certainty that the intervention led to the outcome change. There is currently no gold standard for demonstrating causality in QI projects. However, it has been postulated that the application of the Bradford Hill criteria (BHC) might be of assistance to achieve this end [17]. When applying the BHC to our QI project, it is clear that demonstrating causality could have been improved by having a comparator group (e.g., one which did not receive the leaflet), experimenting with the intervention to see if that changed the results, and collecting more data from a different perspective (e.g., percentage of PDC appointments to review punch biopsies) to demonstrate the consistency of results. However, there are a number of the BHC that we have met. The result finding of 34% ARR and 63% RRR demonstrates a sizeable strength of association. Furthermore, the consistency of this association can be demonstrated by the temporality seen in the run chart. Finally, plausibility, coherence, and analogy have all been demonstrated by other QI projects, which have demonstrated the effectiveness of patient information leaflets [13,14]. We can therefore be fairly confident that the intervention led to the desired results as predicted by the process mapping and driver diagram. In the future, we will endeavor to use comparator groups, statistical processing charts, and alternative metrics to help demonstrate causality.

This study could also have been improved by focusing on balancing measures such as complication rates (e.g., infection or hematoma rates), returns to PDC, and cost of printing to fully explore any issues caused by the intervention. While we are confident in the low complication rate and overall benefit of the intervention, quantitative balancing measure data would be needed to confirm this.

## Conclusions

In this study, we demonstrated that we were able to reduce the number of PDC referrals for patients who had received a punch biopsy by the use of a patient information leaflet. While our focus and approach were narrow, we believe that patient information leaflets can serve to support patients and reduce the NHS outpatient burden. Due to the simplicity of the intervention, it can be spread into many other contexts with concurrent work being done to measure their benefits.

## Figures and Tables

**Figure 2:**
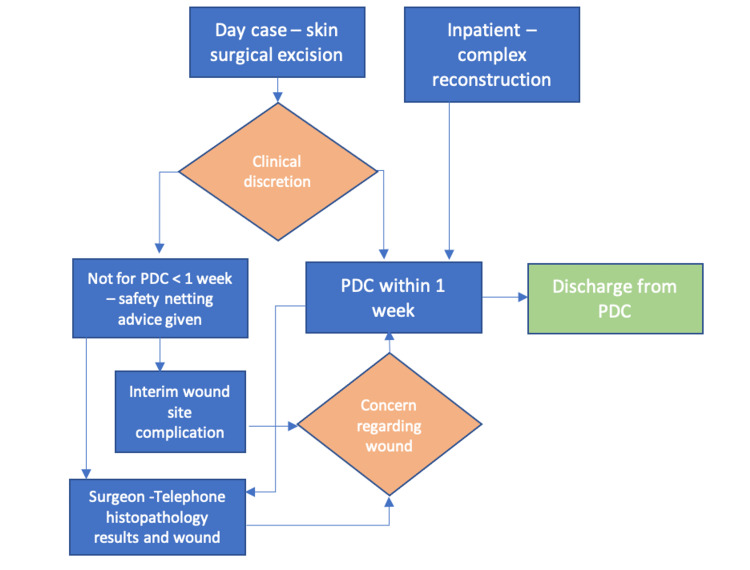
Process map demonstrating patient journey to PDC PDC: plastic dressing clinic

**Figure 3:**
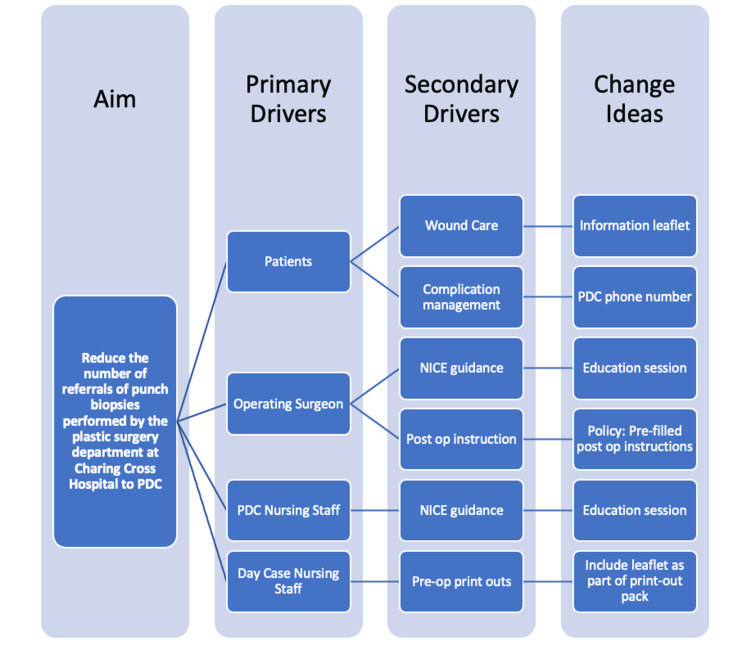
Driver diagram to produce change ideas to achieve the aim PDC: plastic dressing clinic, NICE: National Institute for Health and Care Excellence

**Figure 4:**
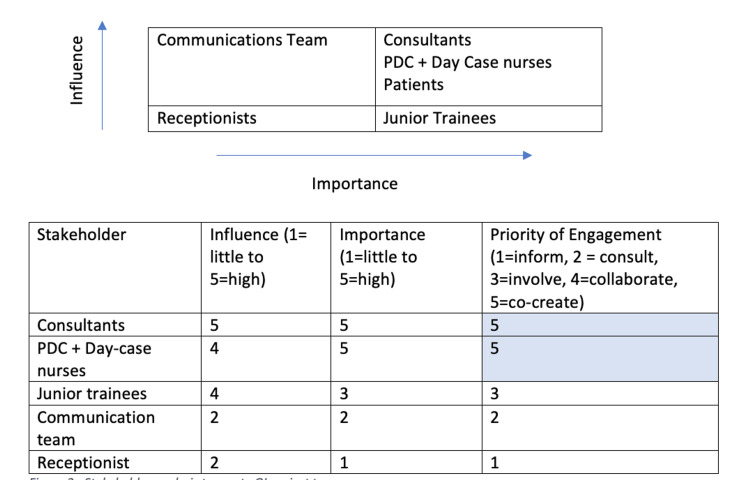
Stakeholder analysis for priority of engagement PDC: plastic dressing clinic

## Appendices

Appendix 1

Figure 2 shows the process map that was created to understand the patient journey from intervention to PDC.

Appendix 2

The driver diagram used to identify change ideas most likely to achieve the aim of reducing punch biopsy wound review in PDC is shown in Figure 3.

Appendix 3

A stakeholder analysis undertaken to identify the key individuals for decision-making and implementation is shown in Figure 4.
